# Electronic phenotyping of community-acquired pneumonia: A tool for inpatient syndrome-specific antimicrobial stewardship

**DOI:** 10.1017/ash.2023.394

**Published:** 2023-09-29

**Authors:** Amy Chang, Annie Bui, David Ha, William Alegria, Marisa Holubar, Brian Lu, Leah Mische, Rebecca Linfield, Kyle Walding, Emily Mui

## Abstract

**Background:** Using patient data from the electronic health record (EHR) and computer logic, an “electronic phenotype” can be created to identify patients with community-acquired pneumonia (CAP) in real time to assist with syndrome-specific antimicrobial stewardship efforts.^1^ We adapted and validated the performance of an inpatient CAP electronic phenotype for antimicrobial stewardship interventions. **Methods:** An automated scoring system was created within the EHR (Epic Systems) to identify hospitalized patients with CAP based on the variables and logic listed in Fig. 1B. We adapted a score used by the Michigan Hospital Medicine Safety Consortium (HMS) to identify patients with CAP, with additions made to improve sensitivity (Fig. 1).^1^ The score can be displayed in a column within the EHR patient list (Fig. 2). We validated the electronic phenotype via chart review of all hospitalized patients on systemic antimicrobials admitted to a medicine team consecutively between November 8 and 18, 2021. Patients who were readmitted within the validation time frame were excluded. We assessed the performance of the electronic phenotype by comparing the score to manual chart review, where “CAP diagnosis” was defined as (1) mention of “pneumonia” or “CAP” as part of the differential diagnosis in the admission documentation, (2) antimicrobials were started within 48 hours of admission, and (3) radiographic findings were suggestive of pneumonia. After initial evaluation, the scoring system was adjusted, and performance was re-evaluated during prospective audit and feedback performed on EHR CAP–positive patients over 13 days between July 2022 and December 2022. **Results:** We included 191 patients in our initial validation cohort. The CAP score had high sensitivity (95.83%), specificity (92.2%), and negative predictive value (99.35%), though lower positive predictive value (63.89%) was noted (Table 2). The rules were further refined to include bloodstream infection only with *Haemophilus influenza* or *Streptococcus pneumoniae* in rule 2B, and azithromycin was removed from “CAP antibiotics.” After these changes, repeated evaluation of 88 patients with positive CAP EHR score was performed, and only 20 (23%) were considered false-positive results. **Conclusions:** Electronic phenotypes can be used to create automated tools to identify patients with CAP with reasonable performance. Data from this tool can be used to guide more focused antimicrobial stewardship interventions and clinical decision support in the future. **Reference:** Vaughn VM, et al. A statewide collaborative quality initiative to improve antibiotic duration and outcomes in patients hospitalized with uncomplicated community-acquired pneumonia. *Clin Infect Dis* 2022;75:460–467.

**Figure:**
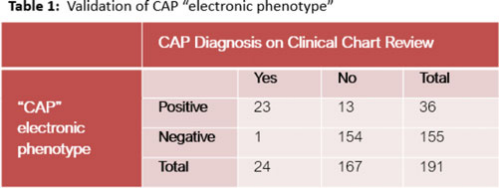

**Figure:**
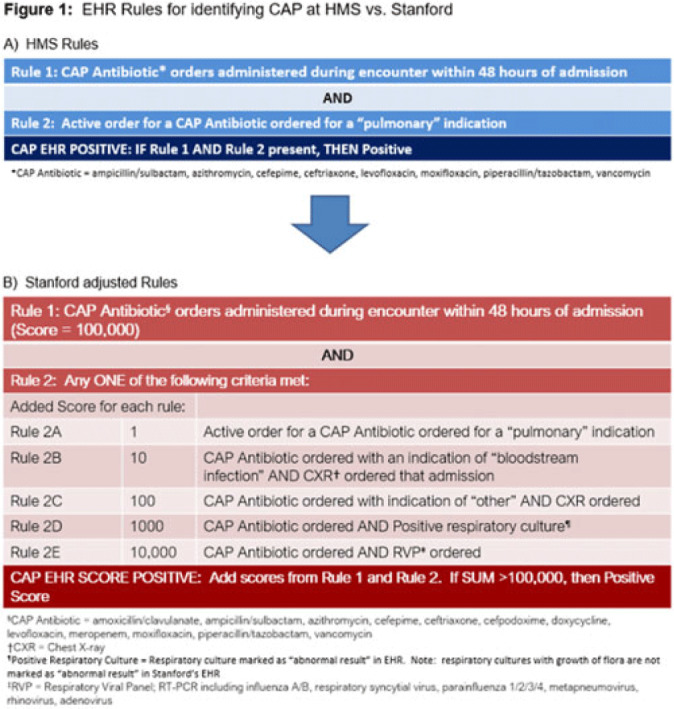

**Figure:**
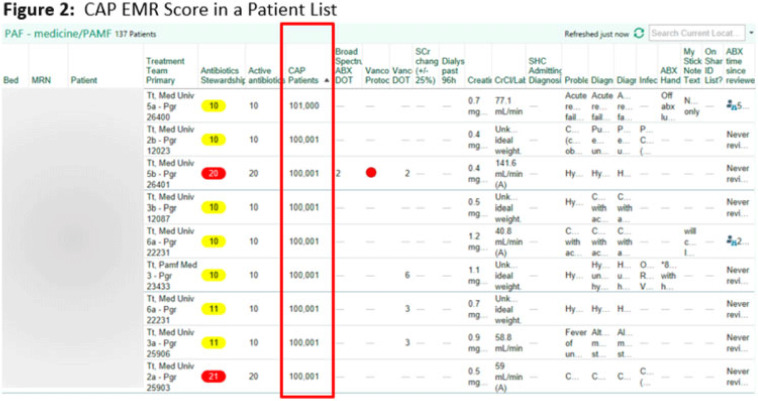

**Disclosures:** None

